# Marked changes in neuropeptide expression accompany broadcast spawnings in the gastropod *Haliotis asinina*

**DOI:** 10.1186/1742-9994-9-9

**Published:** 2012-05-09

**Authors:** Patrick S York, Scott F Cummins, Sandie M Degnan, Ben J Woodcroft, Bernard M Degnan

**Affiliations:** 1 Centre for Marine Science, School of Biological Sciences, University of Queensland, Brisbane, Queensland, 4072, Australia; 2 Faculty of Science, Health, Education and Engineering, University of the Sunshine Coast, Maroochydore, 4558, Australia; 3 Australian Centre for Ecogenomics, University of Queensland, Brisbane, Queensland, 4072, Australia

**Keywords:** Reproduction, Tropical abalone, *Haliotis asinina*, Neuropeptide, Neuromodulator

## Abstract

**Introduction:**

A huge diversity of marine species reproduce by synchronously spawning their gametes into the water column. Although this species-specific event typically occurs in a particular season, the precise time and day of spawning often can not be predicted. There is little understanding of how the environment (e.g. water temperature, day length, tidal and lunar cycle) regulates a population’s reproductive physiology to synchronise a spawning event. The Indo-Pacific tropical abalone, *Haliotis asinina*, has a highly predictable spawning cycle, where individuals release gametes on the evenings of spring high tides on new and full moons during the warmer half of the year. These calculable spawning events uniquely allow for the analysis of the molecular and cellular processes underlying reproduction. Here we characterise neuropeptides produced in *H. asinina* ganglia that are known in egg-laying molluscs to control vital aspects of reproduction.

**Results:**

We demonstrate that genes encoding APGWamide, myomodulin, the putative proctolin homologue whitnin, FMRFamide, a schistosomin-like peptide (SLP), a molluscan insulin-related peptide (MIP) and a haliotid growth-associated peptide (HGAP) all are differentially expressed in the anterior ganglia during the two week spawning cycle in both male and female abalone. Each gene has a unique and sex-specific expression profile. Despite these differences, expression levels in most of the genes peak at or within 12 h of the spawning event. In contrast, lowest levels of transcript abundance typically occurs 36 h before and 24 h after spawning, with differences in peak and low expression levels being most pronounced in genes orthologous to known molluscan reproduction neuromodulators.

**Conclusions:**

Exploiting the predictable semi-lunar spawning cycle of the gastropod *H. asinina*, we have identified a suite of evolutionarily-conserved, mollusc-specific and rapidly-evolving neuropeptides that appear to contribute to the regulation of spawning. Dramatic increases and decreases in ganglionic neuropeptide expression levels from 36 h before to 24 h after the broadcast spawning event are consistent with these peptides having a regulatory role in translating environmental signals experienced by a population into a synchronous physiological output, in this case, the release of gametes.

## Introduction

The Mollusca is an extraordinarily diverse and successful phylum whose members occupy a wide range of terrestrial, freshwater and marine habitats. In the marine environment, molluscs currently account for approximately 23% of the 230,000 known animal species, a proportion rivalled only by the Crustacea [1], and it is estimated that over 50% of extant molluscs still have yet to be discovered and described [2]. The success of marine molluscs can be attributed partially to their varied modes of reproduction, which range from synchronous broadcast spawning entrained by environmental cues (e.g. chitons, bivalves and gastropods) to copulation that is accompanied by species-specific behaviours (cephalopods and gastropods).

Synchronised broadcast spawning of vast numbers of gametes enables extensive mixing of genetically diverse gametes sourced from multiple relatively sedentary individuals. The ensuing pelagic larval phase enables widespread dispersal of offspring, potentially increasing rates of survival and allowing gene flow between geographically-distant populations (e.g. [3-5]). Despite the importance of the synchronisation of spawning in molluscs and other marine animals in maximising fertilisation and dispersal, there currently is little understanding of the controlling mechanisms. Although day length, water temperature, lunar cycle and tidal cycle have all been correlated with gamete release (e.g. [6-11]), only in a few cases have the environmental cues that induce spawning been characterised to a level that spawning events can be predicted with high accuracy [7,12-16]. The endogenous physiological changes that result in the synchronous release of gametes within a population or species remain largely unknown across the animal kingdom.

In molluscs, regulation of the reproductive cycle has been attributed at least in part to various neuropeptides. Investigations primarily in egg-laying species suggest that regulation is achieved not by any single peptide, but rather by a complex cocktail of neurohormones [17-26]. Neuromodulator oligopeptides have specific effects on reproductive organs. For example, APGWamide contributes to peristalsis in the vas deferens of the great pond snail *Lymnaea stagnalis*[17,18]. This hormone also has been implicated as a spawning stimulant in the male tropical abalone *Haliotis asinina*[27]. In *L. stagnalis,* the caudodorsal cell hormone (CDCH) contributes to oocyte release, egg packaging and oviposition [19,28], while the dorsal body hormone (DBH) regulates vitellogenesis and female cell maturation [29]. By comparison, the schistosomin peptide hormone inhibits reproduction by inhibiting CDCH and DBH [20].

Amongst broadcast spawning molluscs, there is less known about hormonal regulation of reproduction. *H. asinina* has a highly predictable spawning cycle, allowing for a detailed analysis of the factors influencing reproduction and spawning. In the wild, *H. asinina* spawns fortnightly in a highly predictable and synchronised manner through the reproductive season, which on the southern Great Barrier Reef lasts about 6 months [7,12]. Spawning is tightly correlated with spring tides associated with new and full moons, with spawning events occurring within an hour of the evening high tide [7]. Final oocyte maturation and release from gonadal connective tissue (trabeculae) occurs about 18 h earlier, around the time of the morning low tide [12]. The timing and height of this low tide has been implicated in controlling the cascade of events that lead to spawning [7]. In addition, abalone removed from natural lunar and tidal cycles (i.e. transported to an indoor closed system away from the sea) maintain synchronous, fortnightly spawnings for at least two cycles (i.e. one month), indicating that endogenous rhythms play a regulating role and that the natural spawning cycle can not be disrupted by removing *H. asinina* from its natural habitat [7].

Here, we describe how *H. asinina* orthologues of four well established molluscan reproduction-related neuropeptide-encoding genes - *APGWamide*, *myomodulin*, *FMRFamide* and *schistosomin* - and three additional neuropeptide genes - *whitnin*, *haliotid growth associated peptide (HGAP), and molluscan insulin-related peptide (MIP)* - vary in expression in the cerebral and pleuropedal ganglia (hereafter collectively referred to as the anterior ganglia) throughout the two week *H. asinina* reproductive cycle. Using quantitative reverse transcription-polymerase chain reaction (qPCR), we compare the expression levels of these genes in male and female anterior ganglia. In the two days prior to and the day following the spawning event, all neuropeptide genes were differentially expressed, with some showing peaks in expression at the spawning event and others 12 h prior to or after the event. In many cases, expression levels differed in male and female ganglia for a given gene. These results are consistent with the neurohormones secreted from anterior ganglia playing a role in controlling a synchronised broadcast spawning event in *H. asinina*.

## Results

### Sequence and post-translational processing of Has-APGWamide, Has-Myomodulin and Has-Whitnin

**Figure 1:**
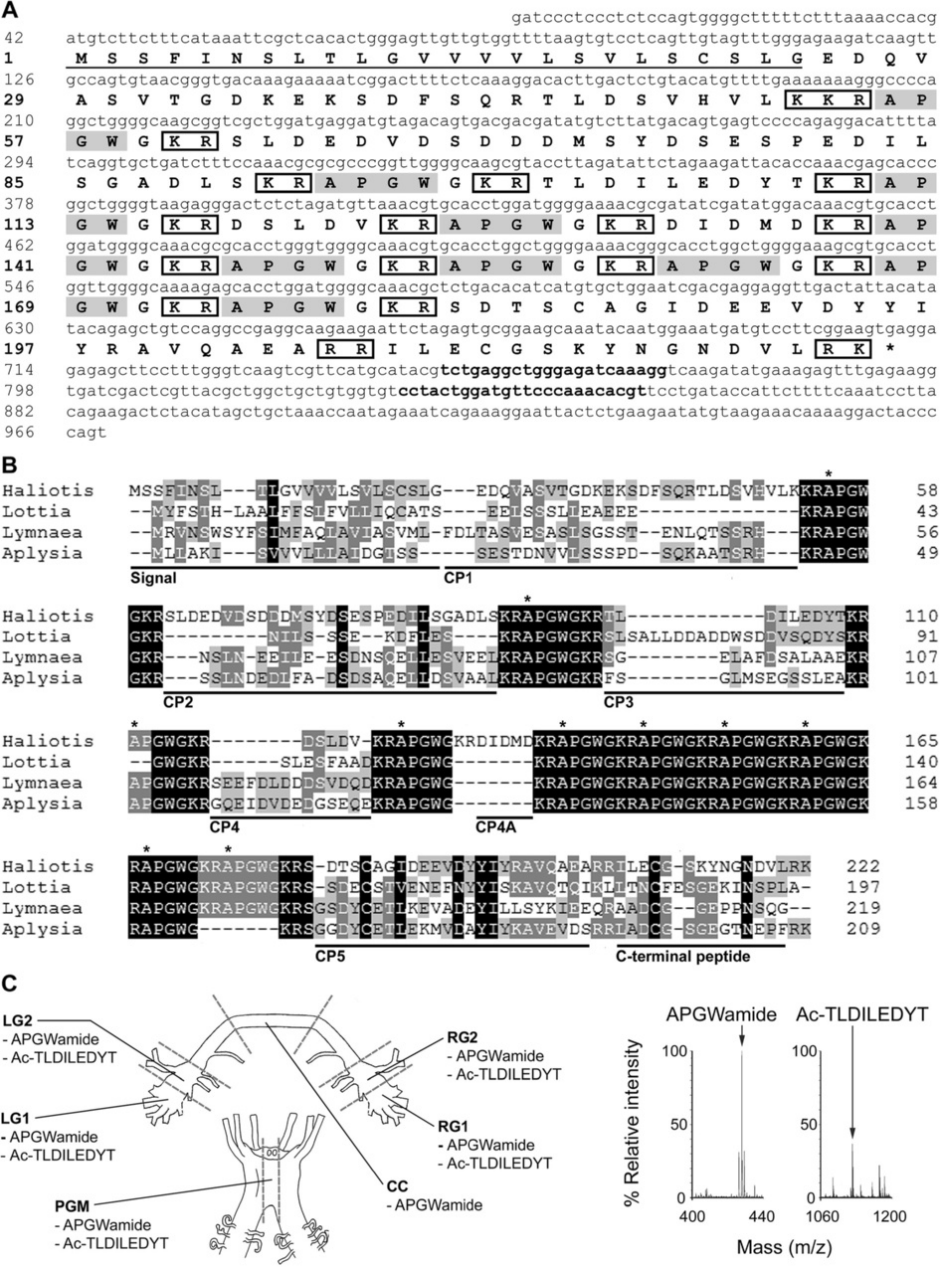
**Characterisation of *Has-APGWamide* .** (**A**) Nucleotide and predicted amino acid sequence of *Has-APGWamide* (nucleotide and amino acids are numbered on left). A predicted signal sequence is underlined, and predicted dibasic and tribasic cleavage site residues are boxed. The *Has-APGWamide* qPCR primers correspond to the nucleotides 750–771 and 830–853, identified here in bold. (**B**) Multiple sequence alignment of molluscan APGWamide prepropeptide sequences. Shading to four levels shows conservation as per Nicholas et al. (1997) [33]. The start of each *H. asinina* APGWamide is indicated with an asterisk. *HaliotisH. asinina* [GenBank:JN606061]; *LottiaLottia gigantea*[34]; *LymnaeaLymnaea stagnalis* ([GenBank:1811269A], [35]); *AplysiaA. californica* ([GenBank: NP_001191561], [30]). (**C**) Schematic representation of *H. asinina* anterior ganglia showing regions analysed by MALDI-TOF-MS. Representative mass peaks were identified that match APGWamide (m/z 428.9) and the acetylated CP3 peptide Ac-TLDILEDYT (m/z 1124.8) (right). Signal, signal sequence; LG1, left cerebral ganglia region 1; LG2, left cerebral ganglia 2; RG1, right cerebral ganglia 1; RG2, right cerebral ganglia 2; CC, cerebral commissure; PGM, pleuropedal ganglia middle; Ac, acetylation; amide, amidation; m/z, mass to charge ratio.

A single, partial-length clone encoding *Has-APGWamide* was identified from the reproductively active anterior ganglia vs. non-reproductively active anterior ganglia (RA/NRA) suppression subtractive hybridisation (SSH) library (see methods), and its sequence was extended by rapid amplification of cDNA ends (RACE). Isolated full-length sequence length was 969 bp [GenBank:JN606061], encoding a 222 residue prepropeptide (Figure 1A). *Has-APGWamide* is predicted to encode 10 copies of APGWamide, 6 connecting peptides (CPs), and a C-terminal peptide, and is most similar to the *Aplysia californica* APGWamide gene [30] (Figure 1B); *H. asinina* CPs are numbered using the system for *Aplysia*[31]. Examination of anterior ganglia sections by matrix-assisted, laser desorption-ionisation time-of-flight mass spectrometry (MALDI-TOF-MS) (Figure 1C.) revealed masses consistent with APGWamide (m/z 428.5) in all sections examined (cf. [32]), and an acetylated CP3 peptide (m/z 1124.2) was found in all sections except the cerebral commissure. By ganglionic region, observed m/z were: left cerebral ganglia section 1 (LG1): APGWamide: 427.7, 428.7, 428.8, 428.9, 429; CP3: 1124.8; left cerebral ganglia section 2 (LG2): APGWamide: 427.7, 428, 428.7, 428.9; CP3: 1124.7, 1124.8; cerebral commissure (CC): APGWamide: 427.7, 428.7; right cerebral ganglia section 1 (RG1): APGWamide: 427.7, 428.7, 428.9, 429, 429.1; CP3: 1124.7, 1124.8; right cerebral ganglia section 2 (RG2): APGWamide: 427.7, 428.7, 428.9, 429, 429.1; CP3: 1124.7, 1124.8; pleuropedal ganglia middle section (PGM): APGWamide: 427.7, 428.7, 428.9, 429; CP3: 1124.8, 1125.1.

**Figure 2:**
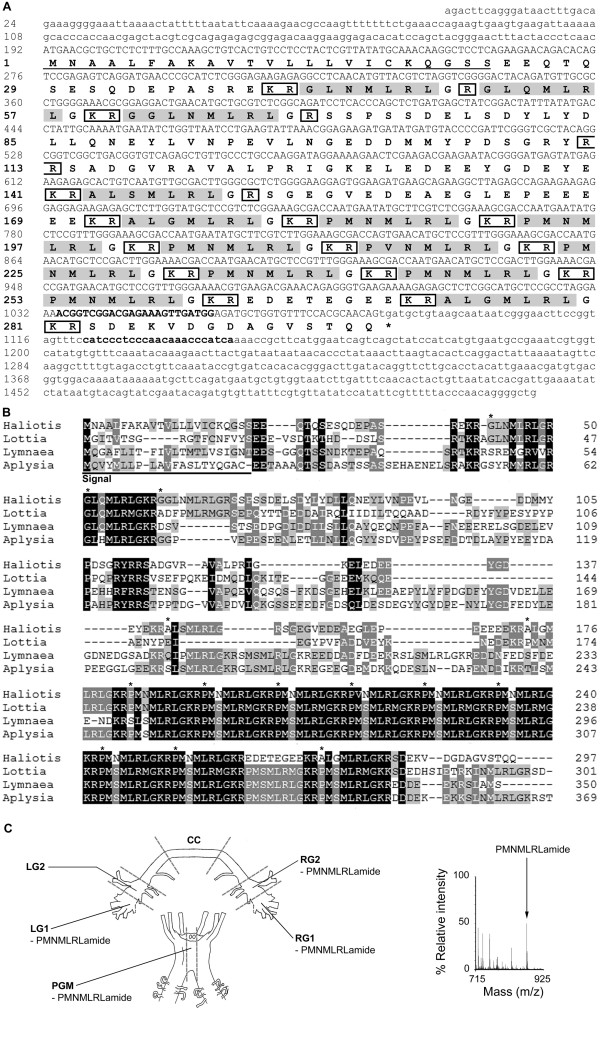
**Characterisation of *Has-Myomodulin* .** (**A**) Nucleotide and predicted amino acid sequence of *Has-Myomodulin* (nucleotide and amino acids are numbered on left). A predicted signal sequence is underlined and predicted monobasic and dibasic cleavage sites are boxed. Predicted myomodulins cleaved from the precursor are shaded. *Has-Myomodulin* qPCR primers described in Table 1 correspond to nucleotides 1034–1057 and 1122–1144, here shown in bold. (**B**) Multiple sequence alignment of molluscan myomodulin prepropeptide sequences. Shading is to 4 levels, and indicates conservation as described in Nicholas et al. (1997) [33]. Asterisks indicate the start of *H. asinina* myomodulins. *HaliotisH. asinina* [GenBank:JN606062]; *LottiaLottia gigantea* (Joint Genome Institute, Protein ID 159404); *LymnaeaLymnaea stagnalis* ([GenBank:X96933]; [39]); *AplysiaA. californica* ([GenBank:S64300], [38]). The C-terminal Q has been removed from the *Aplysia* sequence. (**C**) Schematic representation of *H. asinina* anterior ganglia showing regions analysed by MALDI-TOF-MS. Identification by MALDI-TOF-MS of the most commonly encoded myomodulin PMNMLRLamide in regions of *H. asinina* anterior ganglia. Peak shown is representative of PMNMLRLamide (m/z 872.9) (right). Signal, signal sequence; LG1, left cerebral ganglia region 1; LG2, left cerebral ganglia 2; RG1, right cerebral ganglia 1; RG2, right cerebral ganglia 2; CC, cerebral commissure; PGM, pleuropedal ganglia middle; amide, amidation; m/z, mass to charge ratio.

Two partial-length sequences encoding the *Has-Myomodulin* transcript were found in the RA/NRA SSH library. *Has-Myomodulin* sequence was extended *in silico* by alignment with previously discovered expressed sequence tags (ESTs) ([GenBank:GT277631, GenBank:GT275969, GenBank:GT273228, GenBank:GT067419, GenBank:GT067304]; [36,37]). The isolated final sequence length was 1526 bp (Figure 2A, [GenBank:JN606062]), encoding a well-conserved 297 residue prepropeptide (Figure 2B), which was most similar to the *A. californica* Myomodulin 1 prepropeptide (BLASTp E-value 2e-49) [38]. *Has-Myomodulin* encodes 7 copies of PMNMLRLamide, two copies of ALGMLRLamide, and single copies of each of PVNMLRLamide, ALSMLRLamide, GGLNMLRLamide, GLNMLRLamide, and GLQMLRLamide. Masses matching the most commonly encoded myomodulin PMNMLRLamide (m/z 873.1) were found in the anterior ganglia by MALDI-TOF-MS (Figure 2C). Observed m/z, by section, were: LG1: 872.8; RG1: 872.8, 872.9; RG2: 872.9; PGM: 872.8.

**Figure 3:**
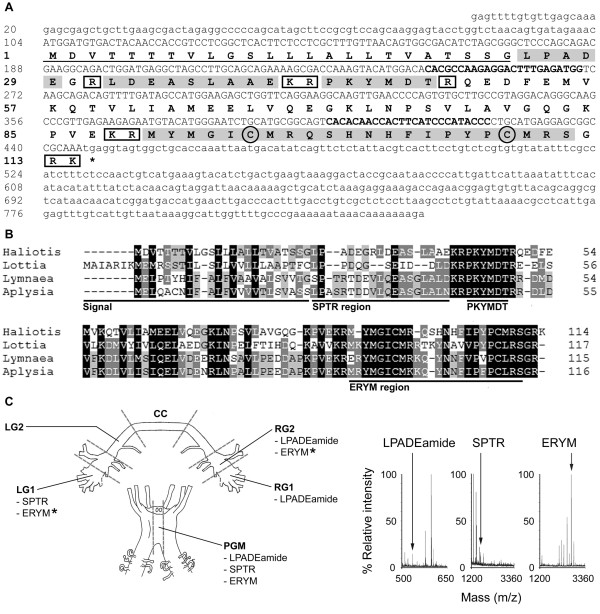
**Characterisation of *Has-Whitnin* .** (**A**) Nucleotide and predicted amino acid sequence of *Has-Whitnin* (nucleotide and amino acids are numbered on left). A predicted signal sequence is underlined. Predicted monobasic and dibasic basic cleavage sites are boxed. Predicted peptides cleaved from the precursor are shaded. Putative disulphide bonded cysteines within the predicted ERYM peptide are circled. Nucleotides 246–269 and 399–423, in bold, correspond to the *Has-Whitnin* qPCR primers. (**B**) Multiple sequence alignment of *H. asinina* Whitnin prepropeptide with molluscan homologues. The 4-level shading indicates conservation, as detailed in Nicholas et al. (1997) [33]. *HaliotisH. asinina* GenBank:JN606063; *LottiaLottia gigantea*[34]; *LymnaeaLymnaea stagnalis* ([GenBank:AAF36485]; [42]); *AplysiaA. californica* ([GenBank:AAV84472]; [41]). (**C**) Schematic representation of *H. asinina* anterior ganglia showing regions analysed by MALDI-TOF-MS. MALDI-TOF-MS detection of predicted *Has-Whitnin* gene products in regions of *H. asinina* anterior ganglia. Asterisks indicate that masses consistent with both disulphide bonded and non-disulphide bonded versions of the predicted ERYM peptide were found in LG1 and RG2. Peaks shown represent LPADEamide (m/z 541.7), SPTR (m/z 1656.6), and ERYM with disulphide bonded cysteines (m/z 2699.9) (right). Signal, signal sequence; LG1, left cerebral ganglia region 1; LG2, left cerebral ganglia 2; RG1, right cerebral ganglia 1; RG2, right cerebral ganglia 2; CC, cerebral commissure; PGM, pleuropedal ganglia middle; amide, amidation; m/z, mass to charge ratio.

The RA/NRA SSH library revealed two partial-length *whitnin* homologues, extended *in silico* using previously discovered EST sequences ([GenBank:GD241801, GenBank:GT276135, GenBank:GT276859]; [37,40]) to produce a final sequence of 833 bp [GenBank:JN606063] that encodes a prepropeptide of 114 amino acids (Figure 3A). Has-Whitnin dibasic cleavage sites and primary structure appear conserved (Figure 3B), with greatest similarity to *A. californica* whitnin [41]. The whitnin gene has previously been referred to as the *SPTR* gene in *Lymnaea stagnalis*[42] and as a *PKYMDT* or *proctolin* gene in *Lottia gigantea*[34]. We select here the name Whitnin to describe the gene and the entire encoded peptide, with SPTR, ERYM and PKYMDT nomenclature here used to describe relevant sub-regions (see Figure 3B). Post-translational processing is predicted to produce peptides including an SPTR homologue, the SPTR derivatives LPADEamide and LDEASLAAE, the conserved PKYMDT peptide proposed as the molluscan homologue of Proctolin [34], and a processed ERYM peptide (Figure 3B). Anterior ganglia examination by MALDI-TOF-MS revealed masses consistent with LPADEamide (m/z 542.6), also its SPTR peptide precursor LPADEGRLDEASLAAE (m/z 1656.8 Da). Further masses matching a post-translationally modified Has-ERYM peptide (MYMGICMRQSHNHFIPYPCMRSamide); both disulphide bonded (m/z 2700.2) and non-disulphide bonded (m/z 2702.3) versions of the same peptide were observed (Figure 3C). By ganglia section, observed masses were m/z: LG1: SPTR: 1656.6; ERYM (cysteines bonded) 2699.9, 2700.9; ERYM (cysteines unbonded): 2702; RG1: LPADEamide: 541.7; RG2: LPADEamide: 541.6, 541.7; ERYM (linked cysteines): 2699.9, 2700; ERYM (unbonded): 2701.9, 2702; PGM: LPADEamide: 542.8; SPTR: 1656.5; ERYM (linked cysteines): 2699.9, 2700.9.

### Neuropeptide genes have dynamic and idiosyncratic expression profiles during the H. asinina spawning cycle

**Figure 4:**
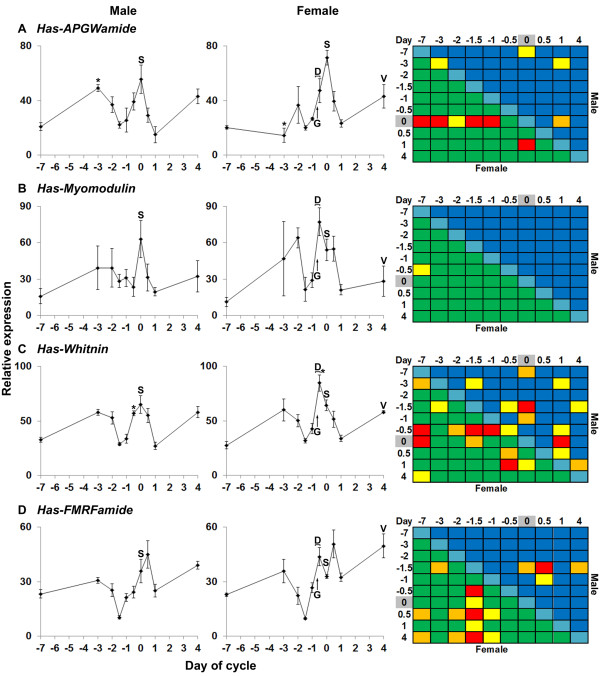
**Quantitative PCR expression profiles of neuromodulators during the *H. asinina* reproductive cycle.** (**A**-**D**) Relative gene expression in anterior ganglia during the reproductive cycle of male and female *H. asinina*. N = 4 anterior ganglia/data points for male or female as relevant. Error bars represent standard error of the mean. Asterisks indicate a significant difference in expression between genders for the indicated timepoint. Notable events during the spawning cycle are indicated: D, dissociation of Cohort I oocytes from trabeculae; G, germinal vesicle breakdown at onset of oocyte maturation; S, time of spawn; V, vitelline envelope appears around the developing, almost full size, Cohort I oocytes. Heat maps (right) indicate level of significance (P < 0.05) for comparisons of gene expression between gender/timepoint groups by Tukey’s HSD test. Background blue cells indicate male, green cells indicate female, aqua cells indicate corresponding time points between males and females. Significant differences in expression are indicated: yellow cells P < 0.05; orange cells P < 0.01; red cells P < 0.001. Day of spawn is highlighted in grey.

**Figure 5:**
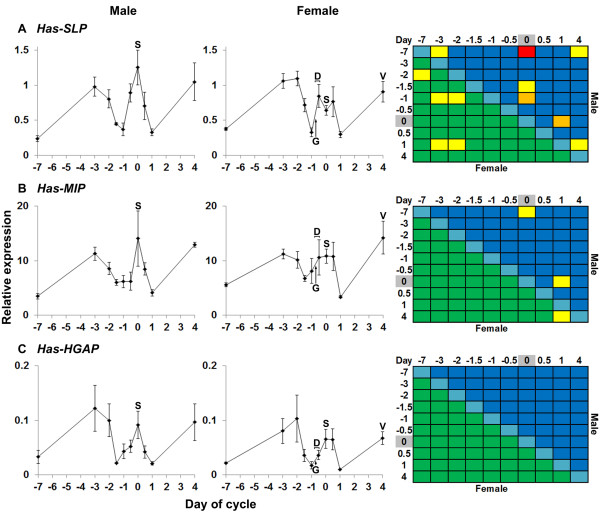
**Quantitative PCR expression profiles of growth-related neuropeptides during the reproductive cycle.** (**A-C**) Relative expression of indicated genes in *H. asinina* male and female anterior ganglia over the reproductive cycle. N = 4 anterior ganglia/data point for male or female as relevant. Error bars display standard error of the mean. Indicated notable spawning cycle events are as per Figure 4. Heat maps (right) indicate significant differences in gene expression between gender/timepoint groups, as per Figure 4.

**Figure 6:**
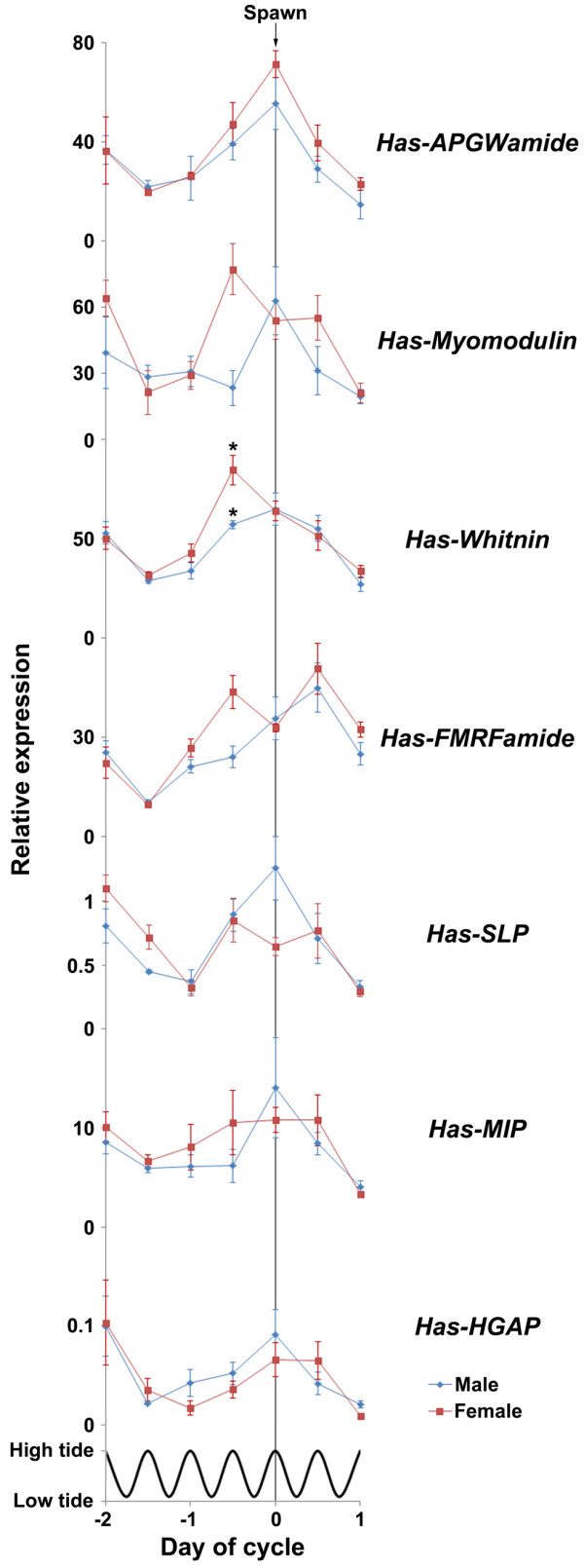
**Comparison of quantitative PCR expression profiles surrounding the spawning event.** Male expression shown in blue (N = 4 anterior ganglia/data point), female expression shown in red (N = 4 anterior ganglia/data point). Error bars display standard error of the mean. Asterisks indicate a significant difference in expression between genders for the indicated timepoint. Tidal cycle is shown; all timepoints were taken at high tide.

To characterise neuropeptide gene expression over the course of the reproductive cycle, we took anterior ganglia from freshly caught groups of male and female *H. asinina* throughout their two week reproductive cycle [7,12]. These samples were collected at the same time each day (2200), except half-day samples, which were collected at 1000. This resulted in all samples taken within 2 days of the spawning event being taken within 1 h 45 min of the high tide. For each time point, 4 gravid males and 4 gravid females were sampled; with 10 time points, 80 *H. asinina* from Heron Island Reef were used in this study. Analysis of transcript abundance of seven neuropeptide-encoding genes - *Has-APGWamideHas-MyomodulinHas-Whitnin, Has-FMRFamide, Has-SLPHas-MIP* and *Has-HGAP* - over the reproductive cycle by qPCR revealed that each gene has a unique and sex-specific expression profile (Figures 4,5,6). In general, expression profiles were consistent across individual males and females for a given gene, although relative gene expression levels did vary between individuals. Standard errors in Figures 4,5,6 depict biological variation.

*Has-APGWamide* transcript abundance peaked on the day of spawning in both males and females (Figure 4A), with all individuals surveyed having remarkably similar expression profiles. Male and female *Has-APGWamide* expression profiles tracked closely throughout the spawning cycle, although males had a secondary prespawning peak in expression on −3 d, while females had a secondary peak on −2 d (Figure 4A; Figure 6), which resulted in a significant difference in expression levels between the sexes on −3 d. There were no detectable differences in male and female expression profiles during the two days leading up to the spawning event, and the day after (Figure 6).

*Has-Myomodulin* expression levels varied the most between individual males and females, and also between sexes (Figure 4B). Male expression levels were maximal at the time of spawning, while female expression levels peaked twice, at −2 d and 12 h before spawning, but because of the large biological variation, one significant difference in expression was observed.

In contrast, changes in *Has-Whitnin* transcript abundance during the semi-lunar spawning cycle were very consistent between individual male and females, with male expression levels reaching maximal levels over a 24 h period surrounding spawning (Figure 4C). As in *Has-Myomodulin*, females had highest expression of 12 h before spawning, around when the oocytes are releasing from the gonadal extracellular matrix.

In both males and females, *Has-FMRFamide* expression increased from the lowest detectable levels at 36 h prespawn to the highest levels at 12 h postspawn (Figure 4D). Unlike males where transcript abundance continually increased during this period, females exhibited a drop in expression at the time of spawning. In both sexes, mRNA levels dropped significantly between 12 and 24 h postspawn.

*Has-SLP* transcript abundance was relatively low overall; lowest levels of expression occurred 24 h pre- and post-spawn in both males and females, with expression increasing at spawning; female expression plateaued over the 24 h period surrounding the spawning event (Figure 5A). *Has-SLP* expression in females was mostly higher during the interspawn period (+1 d to −2 d) than during the time around spawning.

*Has-MIP* expression peaked in males at the time of spawning, while in females it remained relatively constant from −3 d to +0.5 d (Figure 5B). There was notable variation in expression levels between individual males and females, particularly just before, at and after the spawn.

*Has-HGAP* expression levels were also very low throughout the spawning cycle, with relatively high variation in expression between individuals (Figure 5C). As for *Has-SLP* and *Has-MIP*, *Has-HGAP* gene expression levels were not markedly higher over the spawning period compared to the interspawn period.

## Discussion

Unlike most molluscs and broadcast spawning marine invertebrates, *Haliotis asinina* has a highly predictable spawning cycle. Although previous studies established that semilunar synchronous spawning in *H. asinina* is likely the result of tidal entrainment of endogenous rhythms and that these endogenous rhythms persist in captivity for up to six weeks [7,12], the underlying molecular mechanisms controlling this process have not been previously explored. In non-spawning molluscs, neuropeptides secreted from anterior ganglia play a regulatory role in reproduction (e.g. [17-26]). Here we demonstrate that orthologues of these (*APGWamide, myomodulinFMRFamide* and *SLP*) and other (*whitninMIP* and *HGAP*) neuropeptides are differentially expressed in the anterior ganglia of *H. asinina* during the spawning cycle. Although we recognise that this work is essentially correlative in nature and cannot confirm corresponding alterations in peptide production at this time, the described changes in gene expression are consistent with at least some of these genes playing a role in controlling the reproductive physiology and spawning in this gastropod.

### Post-translational processing produces peptides from Has-APGWamide, Has-Myomodulin and Has-Whitnin

Multiple individual peptide neuromodulators are derived from a common propeptide precursor by post-translational cleavage and subsequent modification [43]. As for other *H. asinina* neuropeptides, namely *Has-SLP, Has-MIP, Has-HGAP*[44] and *Has-FMRFamide*[45], we demonstrate here that prohormones encoded by *Has-Myomodulin, Has-APGWamide* and *Has-Whitnin* are processed to yield a repertoire of small neuropeptides. The *Has-Myomodulin* gene encodes 7 copies of the highly conserved PMNMLRLamide, compared to 9 to 10 copies of the corresponding myomodulin in other characterised gastropods [34,38,39]. PMNMLRLamide is present in 4 out of 6 *H. asinina* ganglia regions, indicating widespread anterior ganglia expression.

*Has-APGWamide* encodes 10 APGWamide peptides, similar to the 9 to 10 found in *L. stagnalisA. californica* and *L. gigantea*[31,34,35]. The Has-APGWamide region corresponding to the proposed *A. californica* peptide neurotransmitter CP3 (also known as Cerebral Peptide 1; [30,31] is poorly conserved compared to other molluscan CP3 regions. However, masses consistent with APGWamide and the Has-CP3 peptide (sequence Ac-TLDILEDYT) have been detected by MALDI-TOF-MS, thus supporting our predicted processing model. The detection of APGWamide in all anterior ganglia, and of Has-CP3 in all regions except the CC, indicates that these peptides are widely distributed in the anterior ganglia.

In comparison to *APGWamide* and *myomodulinwhitnin* is relatively poorly studied in molluscs (see [34]); a homologue is present in the annelid leech *Hirudo medicinalis*[46]. The post-translational processing of the *L. stagnalis* whitnin yielded two peptides - SPTR and ERYM - with two further probable intervening peptides [42]. Veenstra (2010) [34] posits that one of those intervening peptides – PKYMDT, which follows the SPTR region - may be the molluscan Proctolin homologue. Encoded peptide positions within the Has-Whitnin propeptide appear to be conserved, although PKYMDT peptide was not detected by MALDI-TOF-MS in the current study.

The *L. stagnalis* SPTR peptide is located between the signal peptide and the first conserved dibasic cut site, and is cleaved from the propeptide in an unmodified form [42]; the SPTR model appears to be conserved (cf. *A. californicaL. gigantea*). The evident Has-Whitnin propeptide cleavage at Arg_31_, and subsequent C-terminal amidation of the resulting LPADEG peptide to form the novel LPADEamide, is detected by MALDI-TOF-MS in 3 out of 6 anterior ganglia regions (RG1, RG2 and PGM). The detection of an uncleaved SPTR peptide in two regions examined (LG1 and PGM) likely represents immature peptide, although we cannot exclude the possibility that the *H. asinina* SPTR peptide is secreted and has a function discrete from that of LPADEamide.

The *L. stagnalis* ERYM peptide is C-terminally amidated, with a single internal disulphide bond [42]. Amidated *H. asinina* ERYM peptide are present in three anterior ganglia (LG1, RG2, and PGM), consistent with the *L. stagnalis* model. The detection of non-disulphide bonded, amidated ERYM versions (LG1 and RG2) likely represents either artifactual reduction of the disulphide bond during MALDI-TOF-MS or an immature form of the ERYM peptide.

### Upregulation of Has-APGWamide, Has-Myomodulin, Has-Whitin and Has-FMRFamide around oocyte maturation or spawning

All seven neuropeptide genes analysed in this study are differentially expressed across the semilunar spawning cycle of *H. asinina*, with most having the lowest levels of expression prior to and after spawning, and high expression levels occurring at, just before or just after spawning (Figure 6). That said, no two gene expression profiles were the same, and expression levels relative to the consistently-expressed reference genes varied markedly, from less than 0.05 times the reference genes (*Has-HGAP*) to nearly 90 times the reference genes (*Has-Whitnin*).

The increase in expression of *Has-APGWamide* at the time of spawning in male and female *H. asinina* is consistent with the established role of APGWamide in molluscan reproduction, particularly its control of muscles in both male and female gonads and sex organs [17,18,47-49]. The peak in *H. asinina* female expression at spawning, taken together with observations in other molluscs, suggests that APGWamide may contribute to the modulation of the induction and regulation of female spawning. Female *H. asinina* spawn with numerous distinct contractions over a period of approximately 5–15 minutes (personal observations). Based on known functions [49], we speculate that APGWamide may be involved in muscle relaxation between contractions. The observed role of APGWamide in triggering spawning in *H. asinina* males [27] may also relate to muscle relaxation between spawning contractions.

*Has-Myomodulin* expression in the anterior ganglia of male and female *H. asinina* varies markedly between individuals, which made it difficult to detect significant differences in expression between stages of the spawning cycle. Nonetheless, anterior ganglia expression peaks at the time of spawning in males and when oocytes dissociate from the female ovary (−0.5 d). Although it is unclear what the precise role of *Has-Myomodulin* has in reproduction, in other molluscs myomodulins have a well-established role in neuromodulating a variety of reproductive processes [21,23,25]. Myomodulins also have a well-documented role in feeding [50-55]. Interestingly, we have previously shown that expression of *Has-Myomodulin* in the anterior ganglia is also correlated with feeding status in aquaculture [36]. Indeed, variation in individual foraging success may explain the high individual variation in expression of this gene in this study. However, as all animals were collected from the wild, pre-experiment feeding regimes were not known.

We observed many significant differences in *Has-Whitnin* gene expression over the reproductive cycle in both males and females. As the relative expression level of *Has-Whitnin* correlates tightly with the stage of the spawning cycle, this gene may be an excellent expression marker for the spawning cycle. Given the established neuromodulatory role of whitnin-derived peptides [34,42,56], we suggest that *Has-Whitnin* contributes to the control of reproduction. *Has-Whitnin* expression in female *H. asinina* peaks 12 hours prior to spawning, at which time oocytes release from the gonadal trabeculae [12]. In contrast, male expression peaks at time of spawn. These and other sex-specific differences in *Has-Whitnin* gene expression in the reproductive cycle suggest a gender-specific role for this gene.

FMRFamide is widely distributed in the animal kingdom. Its function is diverse, and includes modulation of feeding behaviour [57], retinal response to light [58], sexual maturation [58], apoptosis [59], osmoregulation [60-63], and regeneration [64-66]. *Has-FMRFamide*[45] expression levels change dramatically over the semilunar spawning cycle, and between males and females. The gender-specific reproductive roles of FMRFamide in closely related molluscs [17,67-72] provides a compelling argument that *Has-FMRFamide* is a reproduction-related gene. In particular, the peak in female *Has-FMRFamid*e expression at −0.5 d, followed by a notable reduction at time of spawn, is consistent with known reproductive roles of FMRFamide in other molluscs.

FMRFamide has a demonstrated role in the inhibition of egg-laying in the molluscs *L. stagnalis* and *A. californica*; it prevents secretion of the hormones that trigger egg laying [67,69]. We suggest that the peak in *Has-FMRFamide* expression at Day −0.5 may reflect *H. asinina* use of FMRFamide to inhibit precocious spawning. The subsequent drop in female expression at time of spawn, which is highly consistent among individuals, is consistent with this proposition. Such FMRFamide-mediated inhibition of gamete release has so far been demonstrated to pertain only to females. Indeed, given that *L. stagnalis* and *A. californica* are hermaphroditic and produce male and female gametes at different times [9,10], it can be assumed that there exist discrete mechanisms for male and female gamete release. The presence of a female peak and lack of a corresponding male peak in *Has-FMRFamide* expression at Day −0.5 is therefore consistent with the notion that FMRFamide is a functionally conserved temporal regulator of female, but not male, gamete release in gastropods. There is a peak in both male and female FMRFamide expression at 0.5 d, which may reflect a role for FMRFamide in recovery from spawning and regeneration of the gonad. In females, phagocytosis of unspawned mature oocytes and the rebuilding of trabeculae are underway at this time [12]. FMRFamide is known to be involved in regenerative processes, including the acceleration of healing [64,65], stimulation of protein and nucleic acid synthesis [73], neural regeneration [66], and the regulation of apoptosis [59].

The biological significance of the marked and transient decrease in *Has-FMRFamide, Has-APGWamide, Has-Myomodulin* (female only), *Has-Whitnin* and *Has-HGAP* (male only) expression levels 36 h before spawning (Figure 6) is unknown. This general decrease in expression corresponds to the morning high tide the day before the spawning event and may signify some time-keeping mechanism linked to a threshold tidal level and endogenous rhythms, as previously proposed [7]. In the next 24 h, the oldest cohort of oocytes in the ovary will undergo germinal vesicle breakdown and dissociate from ovary trabeculae, ready to be spawned [12]. Interestingly, a consistent drop in gene expression in these and the other genes occurs one day after the spawn (Figure 6) suggesting that this might be another time-keeping event, triggering the synchronisation of oogenesis and possibly spermatogenesis for the next spawning event in approximately two weeks. The maintenance, for at least a month, of the synchronous spawning cycle in *H. asinina* that have been removed from the natural environment [7] indicates endogenous signals can maintain rhythmicity without exposure to tide or lunar cycle.

### Has-SLP, Has-MIP and Has-HGAP exhibit variable but low expression levels during the spawning cycle

*Has-SLP, Has-MIP* and *Has-HGAP* exhibit expression profiles similar to that observed for the other neuropeptide genes, with lowest levels of expression tending to be 2 days before and a day after the spawning event (Figure 6). However, *Has-SLP, Has-MIP* and *Has-HGAP* are expressed at much lower levels than the other four genes, and do not exhibit such large differences in transcript abundance between spawning and interspawning stages. Together, these observations are consistent with these three neuropeptide genes having a less important role in controlling spawning.

Schistosomin inhibits molluscan reproduction by inhibiting the production and secretion of female reproductive hormones [20,74-77]. This role appears to be conserved in gastropods that copulate (*L. stagnalisL. ovataBiomphalaria glabrataBiomphalaria pfeifferi*, and *Bulinus truncatus*) [20,74-79]. Other non-reproductive roles for schistosomin also have been postulated, including promotion of growth via triggering the secretion of MIP [20,79] and in larval development [80,81]. In *H. asinina*, a gene encoding a schistosomin-like peptide (*Has-SLP*) was recently isolated and found to be upregulated in fast-growing juvenile abalone [44]. The high expression of *Has-SLP* in the interspawn period reported here is consistent with a conserved role for Has-SLP in the inhibition of female reproductive processes. Based on expression profiles (Figure 6), *Has-SLP* may play a different role in males than in females, although details of this are unknown at this time.

Molluscan insulin-related peptides (MIPs) are molluscan peptide hormones thought to promote growth and regulate nutrient uptake [19,20,82-86]. There is an established relationship between the reproduction-related hormone schistosomin and MIP release: *L. stagnalis* schistosomin causes MIP release, resulting in growth of the animal [20]. However, we have found that the *Has-MIP* gene is down-regulated in fast growing animals compared to slow growers [44]. In the present study, *Has-MIP* expression did not vary markedly through the spawning cycle, although there was a decrease in transcript abundance at −1.5 and +1 d, as observed for many of the other genes. Interspawn expression levels were high or similar to the expression levels around the time of spawning, suggesting a minor role for this peptide in the control of spawning

 The HGAP gene was first found in a *H. asinina* anterior ganglia SSH library created from well-nourished against food-deprived animals ([36]; [GenBank:GT067343]). Further characterisation has revealed that *Has-HGAP* encodes a double-chain secreted peptide and is expressed in all tissues examined except the gill [44]. To date, HGAP has only been found in *H. asinina*. Although it is unknown whether Has-HGAP actively promotes growth as a regulatory hormone, *Has-HGAP* expression is upregulated in fast growing *H. asinina* compared to slow growing individuals [44]. *Has-HGAP* expression overall is the lowest of all genes surveyed in this study and, as for *Has-MIP*, is highest during interspawn periods. In the case of both these genes, there was marked individual variation in expression levels, rendering it difficult to infer much from these expression profiles.

## Conclusions

We report here the sequences of the neuromodulator-encoding genes *Has-APGWamide*, *Has-Myomodulin* and *Has-Whitnin*, and describe the post-translational processing of their encoded peptides. Comparisons to molluscan orthologues indicate that processing of the proneuropeptides is well conserved in all cases. QPCR analysis reveals that expression in the anterior ganglia of *Has-APGWamide, Has-Myomodulin* and *Has-Whitnin*, as well as another neuromodulator-encoding gene *Has-FMRFamide*, varies across the *H. asinina* reproductive cycle. Peaks in the expression levels of these genes correspond to events associated with spawning, including oocyte maturation and dissociation from the ovary. Perhaps most notably, *Has-FMRFamide* expression, along with *Has-APGWamide*, *Has-Myomodulin* and *Has-Whitnin*, is suggestive of a synchronisation mechanism in the reproductive cycle, 36 hours prior to spawning. The temporal map of expression of the seven candidate reproduction-related genes provided in this study will assist future endocrinological studies into molluscs and other marine invertebrates that synchronously release their gametes in a broadcast spawning event. In particular, this study presents a range of neuropeptide candidates to investigate the control of spawning in species with less tractable and predictable spawning, including those of commercial importance.

## Materials and Methods

### Animals

Adult male and female *H. asinina* (gravid and ranging from approximately 12 to 20 cm in length) that were used for the gene expression analyses were collected from Heron Island Reef (Queensland, Australia) under permit, and kept in flow-through seawater tanks with water obtained from the reef flat from where the abalone were collected. For the analysis of gene expression during the spawning cycle, abalone were collected no more than three days before sacrificing. Animals used for peptidomic analyses were collected from Heron Island reef and transported to Bribie Island Research Centre (Queensland Primary Industries and Fisheries, Department of Employment, Economic Development and Innovation), and kept in an inside tank with a 12-hour light/dark cycle. Collected animals were fed to satiety on local algae from Heron Island Reef. Animals housed at Bribie Island Research Centre were fed to satiety with *Gracillaria edulis*, and an artificial food purchased from Adam & Amos Abalone Foods Pty. Ltd. [87].

### Sequence isolation, extension, identification and analysis

RNA isolation, cDNA synthesis and amplification, SSH, cloning, sequencing, and *in silico* sequence extension were carried out as described in York et al. 2010 [36]. SSH utilised anterior ganglia from two reproductively active (RA) and two non-reproductively active (NRA) adult *H. asinina* as Tester and Driver samples, respectively. Where appropriate, the SMART™ RACE cDNA Amplification Kit (Clontech, Mountain View, California) was used to obtain entire coding sequence, as per manufacturer’s protocols.

To identify related sequences, a BLASTx search against the NCBI database [88,89] was performed, with a stringency cutoff e-value of 10^-6^. Neuropeptide post-translational processing was predicted from translated sequence using the NeuroPred [90,91], SignalP [92,93] and SIG-Pred [94] programs. Multiple sequence alignments were done with the Molecular Evolutionary Genetics Analysis software version 4.0 program (MEGA4) [95], using the ClustalW algorithm [96]. Shading of multiple sequence alignments was performed using GeneDoc Version 2.7.000 [33].

### Matrix-Assisted Laser-Desorption/Ionisation-Time of Flight Mass Spectrometry (MALDI-TOF-MS)

MALDI-TOF-MS was performed as described in Cummins et al. (2009) [32]. Briefly, anterior ganglia were removed from 14-month old *H. asinina*, rinsed in aqueous MALDI-TOF-MS matrix solution [20 mg/ml 2,5-dihydroxybenzoic acid (Sigma-Aldrich, St. Louis, Missouri) in 30% acetonitrile/0.1% trifluoroacetic acid], sectioned, and desheathed. Each section was then torn into tiny fragments in matrix solution using dissection forceps. Tiny fragments (< 1 mm) of each section were placed on a MALDI-TOF-MS plate in 0.5 μL matrix solution. A Voyager-DE STR Biospectrometry Workstation (Applied Biosystems, Foster City, California), with N_2_ laser and pulsed ion extraction accessory was used to analyse the fragments, with 500 shots in reflectron mode. Masses were taken as identified if within 1 Da of predicted average mass.

### Reproductive cycle expression analysis of selected genes by qPCR

For each point in the time series, anterior ganglia were dissected from 4 anaesthetised male and female abalone, each which was haphazardly chosen from the pool of previously collected gravid adults, and stored in RNALater at −80°C as previously described [36]. Total RNA was extracted using Tri Reagent (Sigma-Aldrich, St. Louis, Missouri), and reverse transcribed using SuperScript III Reverse Transcriptase (Invitrogen, Carlsbad, California), as per manufacturers’ instructions. Oligonucleotide primers for qPCR were either those used in previous studies [36,44], or were designed using the Primer3 program [97,98] (Table 1). Relative transcript abundance in anterior ganglia was measured between male and female time points taken throughout the course of the 14-day *H. asinina* reproductive cycle using a Roche Lightcycler® 480 II qPCR machine with Lightcycler® 480 SYBR Green I Master (Roche, Penzberg, Upper Bavaria). The reference genes *Has-UEST1506Has-NACA*, and *Has-Ubiquitin* were selected from a pool of potential reference genes [40,44] using the geNorm program [99]. Reference gene validity was also manually confirmed by visual examination of non-normalised, raw qPCR expression data. Normalisation of expression was done using the Relative Expression Software Tool for Rotor-Gene 3000 and 6000, Version 3 [100]. To authenticate differences in expression between gender/time point groups, two-way ANOVA and Tukey’s HSD tests were performed using the R program [101], with P-values of 0.05 or lower taken as significant.

**Table 1:** Oligonucleotide primers for qPCR

**Target gene**	**Primers**	**Tm**
	**(F = forward and R = reverse)**	
*Has-APGWamide*	**F:** TCTGAGGCTGGGAGATCAAAGG	68°C
	**R:** ACGTGTTTGGGAACATCCAGTAGG	
*Has-Whitnin*	**F:** CACGCCAAGAGGACTTTGAGATGG	68°C
	**R:** GGGTATGGGATGAAGTGGTTGTGTG	
*Has-Myomodulin*	**F:** ACGGTCGGACGAGAAAGTTGATGG	68°C
	**R:** TGATGGGTTTGTTGGGAGGGATG	
*Has-FMRFamide*	**F:** TTCGGGAAGCGAGATTCTGGTG	68°C
	**R:** GGTGGATGTGAAACAGCGAACAGTC	
*Has-SLP*	**F:** TCGTCCTCATCGCTCTCGTTGTT	69°C
	**R:** GGAGCCGTTGGAAATGCAGAAG	
*Has-MIP*	**F:** CAAGCGGACGAGTGAACAAGG	67°C
	**R:** TTGCTCTTACAGCGTCTAGCATGG	
*Has-HGAP*	**F:** CTGTGCATTCTCCTTGTCGTAGTCG	68°C
	**R:** GCTCATGTAGCCGAATGATTCTTCC	
**Reference genes**		
(Williams et al., 2009)		
*Has-Ubiquitin*	**F:** TGGCAAGCAGTTGGAAGATGGT	57°C
	**R:** CAGTTGTACTTGGAGGCCAGGAT	
*Has-NACA*	**F:** TGTCGCAAGCCAACGTTTCA	57°C
	**R:** GACAGCATGTTCAGCACTGGT	
*Has-DAu1506*	**F:** AGATGCGTGTATGCTGGAGT	57°C
	**R:** TGGGTACATGCCAATGCT	

### Availability of supporting data

The data sets supporting the results of this article are included within the article.

## Abbreviations

bp, base pair; CC, Cerebral commissure; CDCH, Caudodorsal cell hormone; cDNA, complementary DNA; d, day/s; Da, Dalton; DBH, dorsal body hormone; EST, Expressed sequence tag; HGAP, Haliotid growth-associated peptide; h, hour; Has, Haliotis asinina; LG1/2, left cerebral ganglia section 1/2; MALDI-TOF-MS, matrix-assisted, laser desorption-ionisation time-of-flight mass spectrometry; min, minute; MIP, molluscan insulin-related peptide; m/z, mass to charge ratio; PGM, pleuropedal ganglia middle section; qPCR, quantitative reverse transcription-polymerase chain reaction; RACE, rapid amplification of cDNA ends; RA/NRA, reproductively active anterior ganglia vs. non-reproductively active anterior ganglia; RG1/2, right cerebral ganglia section 1/2; s, second; SLP, schistosomin-like peptide; SSH, suppression substractive hybridisation.

## Competing interests

The authors declare that they have no competing interests.

## Authors’ contributions

Project conception and sampling: S.Y., B.M.D., S.M.D. and S.C.; Gene characterisation: S.Y.; Proteomics: S.Y. and S.C.; Bioinformatics and phylogenetics: S.Y. and B.W.; Writing: S.Y., B.M.D., S.M.D. and S.C. All authors read and approved the final manuscript.
